# Molecular characteristics of hepatocellular carcinomas from different age groups

**DOI:** 10.18632/oncotarget.21353

**Published:** 2017-09-27

**Authors:** Celina Ang, Anthony Shields, Joanne Xiu, Zoran Gatalica, Sandeep Reddy, Mohamed E. Salem, Carol Farhangfar, Jimmy Hwang, Igor Astsaturov, John L. Marshall

**Affiliations:** ^1^ Department of Medicine, Hematology/Oncology, Icahn School of Medicine at Mount Sinai, New York, NY, USA; ^2^ Department of Oncology, Molecular Imaging & Diagnostics Program, Karmanos Cancer Center, Wayne State University, Detroit, MI, USA; ^3^ Department of Medical Affairs, Caris Life Sciences, Phoenix, AZ, USA; ^4^ Department of Pathology, Caris Life Sciences, Phoenix, AZ, USA; ^5^ Hematology/Oncology, Lombardi Comprehensive Cancer Center, Georgetown, University, Washington, DC, USA; ^6^ Levine Cancer Institute, Carolinas Healthcare System, Charlotte, NC, USA; ^7^ Department of Hematology/Oncology, Fox Chase Cancer Center, Philadelphia, PA, USA

**Keywords:** hepatocellular carcinoma, age differences, multiplatform profiling, pathogenic mutations

## Abstract

While most patients in Western countries who are diagnosed with HCC are in their 50s and 60s, HCCs diagnosed at extremes of the age spectrum (i.e., < 40 years and ≥ 75 years) are less common and have been linked with distinct geographic locations and etiologies. Using multiplatform profiling, we identified differences in genetic alterations and protein expression in different age groups within a large cohort of HCC patients (N = 421). Young adult HCC patients (18-39 years’ old) were more likely to be female, living in the West and Midwestern United States, and showed decreased androgen receptor, drug resistance and pro-angiogenic protein expression compared to older patients. *TP53* mutations were the most frequent alteration in young adults (19%), whereas *CTNNB1* mutations occurred in 30-33% of patients ≥ 40 years’ old. The overall frequency of pathogenic and presumed pathogenic mutations was observed to increase significantly with advancing age. To our knowledge, these data represent one of the only studies to analyze age-specific molecular profiles in HCC, and provide a basis for further exploration and validation of these findings with respect to their clinical and therapeutic implications.

## INTRODUCTION

Hepatocellular carcinoma (HCC) is an aggressive malignancy, representing the second and sixth leading cause of cancer deaths worldwide in men and women, respectively [1]. HCC typically arises in the context of chronic liver disease and cirrhosis due to viral hepatitis, excessive alcohol consumption, fatty liver disease and other risk factors. While liver transplantation and resection offer the possibility of long-term survival, advanced HCC is uniformly lethal and there are limited treatment options.

The majority of HCC patients in the Western hemisphere are diagnosed in their 50s and 60s, though HCCs diagnosed at extremes of the age spectrum (i.e., < 40 years, ≥ 75 years) also occur. The highest incidence of HCC in individuals ≥ 75 years' old, particularly in men, has been reported in low-risk Western countries, South Africa and Egypt where hepatitis C prevails [2]; whereas young adult patients (i.e., 20-40 years’ old) diagnosed with HCC primarily reside in hepatitis B endemic regions. In one study, over 85% of patients ≤ 40 years of age were found to be hepatitis B surface antigen seropositive compared to 60% of patients > 40 years (p=0.003), underscoring the strong association between hepatitis B and young onset HCC [3]. East Asian countries have an HCC prevalence in young adults ranging from 0.9-10.9% [3]. Among African Blacks with hepatitis B, 43% are diagnosed with HCC before 40 years of age [4]. Apart from etiologic and geographic differences, younger individuals with HCC tend to present with a higher serum α-fetoprotein and more advanced stage at diagnosis [3, 5–8]. In contrast, HCC in the elderly has been linked with female sex, chronic hepatitis C or no history of viral hepatitis, and a higher prevalence of normal liver than younger patients [9]. Comparisons of age-specific differences in survival have yielded conflicting results with some studies reporting poorer outcomes in younger vs older patients and others reporting no difference [3, 6, 10, 11–15]. To our knowledge, there have not been any studies that have evaluated molecular differences in HCCs between young and elderly patients. We sought to ascertain differences in molecular profiles of HCC patients across the age spectrum and their potential therapeutic implications.

## RESULTS

### Demographics

**Figure 1:**
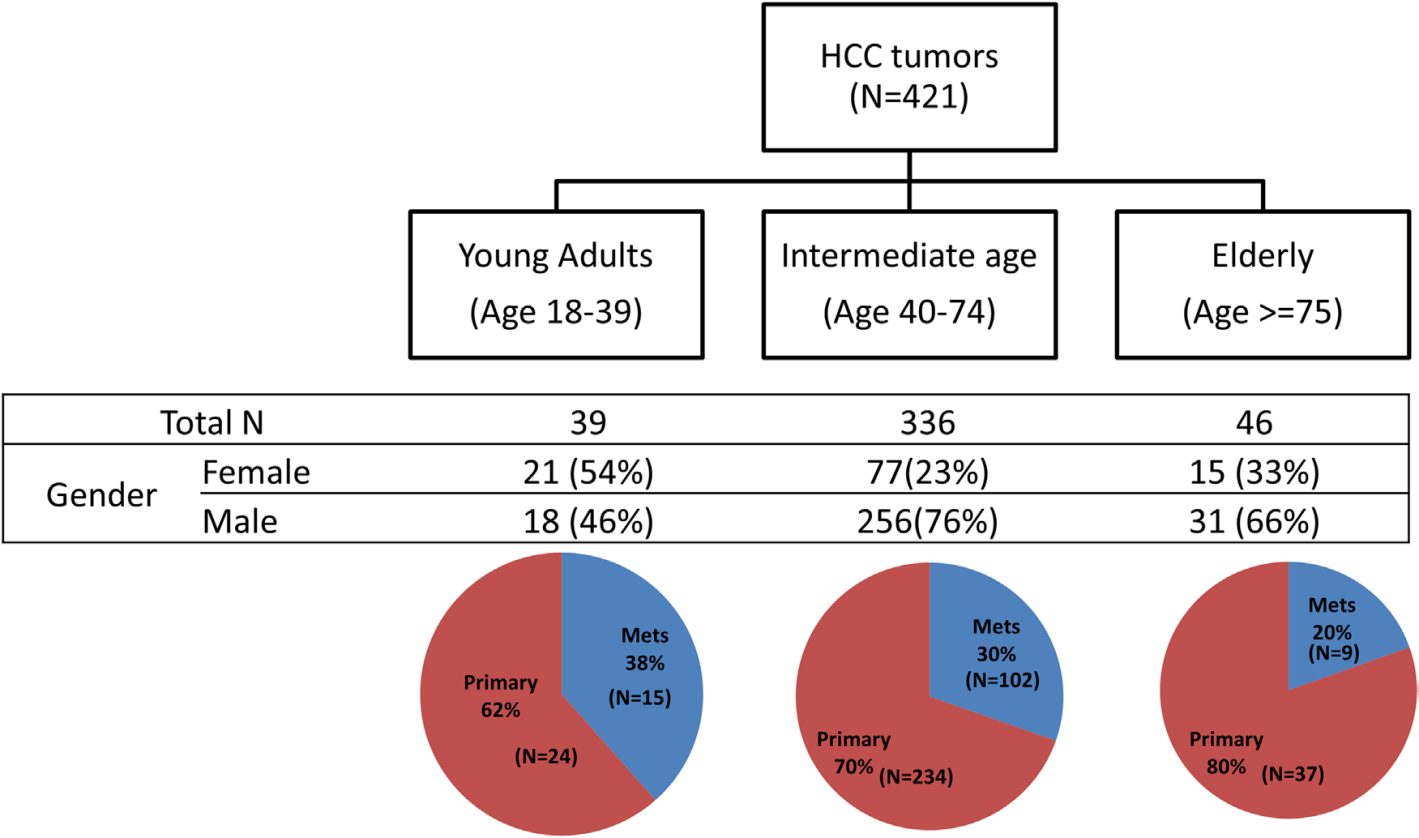
Study cohort composition

A total of 421 specimens were included in this dataset: 39 (9%) were from young adults (18-39 years’ old), 46 (11%) from elderly patients (≥ 75 years’ old) and 336 (80%) from the intermediate age group (40-74 years’ old). There was a significantly higher proportion of females in the young adult HCC subgroup (54%) compared to intermediate age (23%, p<0.0001) and elderly patients (33%, p=0.048). HCC metastatic to another organ comprised 38%, 30% and 20% of the young adult, intermediate age and elderly cohorts, respectively, but these differences were not significant. The proportion of HCC metastases by age group is illustrated in Figure 1 and the breakdown of metastatic sites is shown in Table 1. The most frequently sampled metastatic sites were lymph nodes in young adults and the lungs in the intermediate age group. There was no significant difference among the groups with regards to the use of primary or metastatic tumor for these analyses. Therefore, the subsequent comparisons performed on the tumor groups stratified by age consisted of both primary and metastatic tumors.

**Table 1:** Sites of HCC metastases in different age groups

Metastatic site	Young adult (N=15)	Intermediate age (N=102)	Elderly (N=9)
Lymph nodes	6	13	2
Lung	4	23	1
Peritoneum	2	8	2
Bone	2	17	0
Connective tissue	1	18	2
Brain	0	4	0
Other	0	19	2

**Figure 2:**
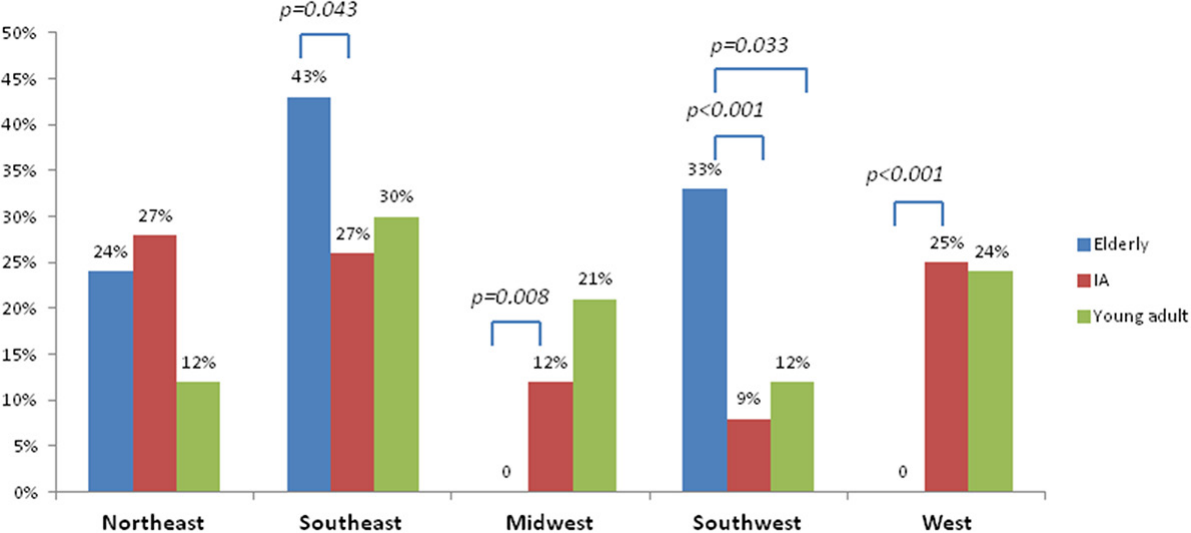
Breakdown of United States region of residence by age

Information on geographic region of residence was available on 398 patients. There were 12 patients from Israel, France, China, Russia, Lebanon, the United Kingdom, Australia and Belgium. Amongst patients living in the United States, the majority resided in the Southeast (29%) followed by the Northeast (25%), West (22%), Midwest and Southwest (12% each). Elderly patients were more prevalent than younger patients in the Southeast and Southwest, whereas patients < 75 years' old were more prevalent in the West and Midwest than elderly patients. The breakdown of region of residence by age amongst patients living in the United States is illustrated in Figure 2.

### Protein expression

**Figure 3:**
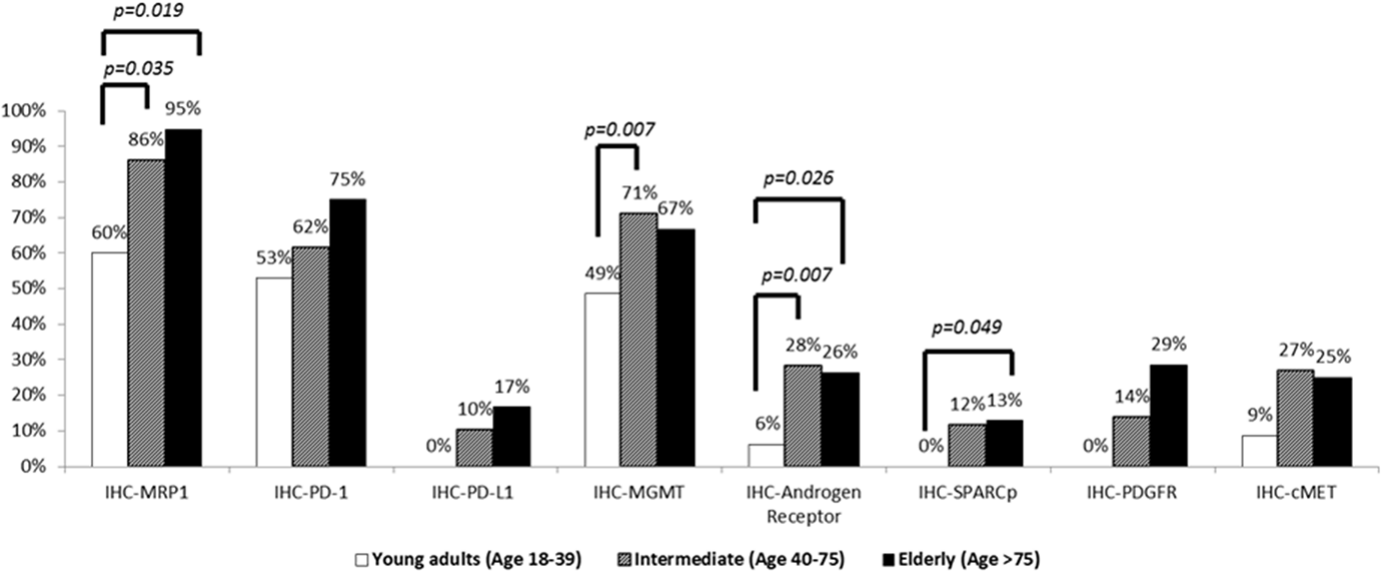
Comparison of protein expression by immunohistochemistry across age groups

As shown in Figure 3, androgen receptor (AR) expression was significantly less frequent in young adults (2/33, 6%) compared to the elderly (10/38, 26%, p=0.026) and intermediate age groups (71/251, 28%, p=0.007). Focusing on male patients, AR expression was significantly less common in young adults (1/16, 6%) than in the intermediate age (67/193, 35%, p=0.019) and elderly (8/25, 32%, p=0.052) groups. In females, there was also a trend towards increased AR expression with advancing age: young adults (1/17, 6%), intermediate age (4/58, 7%) and elderly patients (2/13, 15%, p>0.05).

MGMT expression was significantly more common in intermediate age than young adult patients [71% (190/267) vs 49% (17/35), p = 0.007], and was also more frequent in the elderly subgroup (26/39, 67%). There were significant differences in MRP-1 expression in HCCs in young adults (6/10, 60%) compared to intermediate age (80/93, 86%, p=0.035) and elderly patients (18/19, 95%, p = 0.019). SPARC expression was absent in young adult HCCs but was present in 12% (25/214) and 13% (4/31) of tumors from intermediate age and elderly patients, respectively. PDGFR and PD-L1 were expressed in intermediate age [14% (5/36) and 10% (7/68), respectively] and elderly patients [29% (2/7) and 17% (1/6), respectively], but not young adults [0% (0/7) and 0% (0/7), respectively]. The hepatocyte growth factor receptor c-Met was expressed in over 25% of patients ≥ 40 years' old compared to only 9% of young adults. None of these differences were statistically significant.

### Next generation sequencing

**Figure 4:**
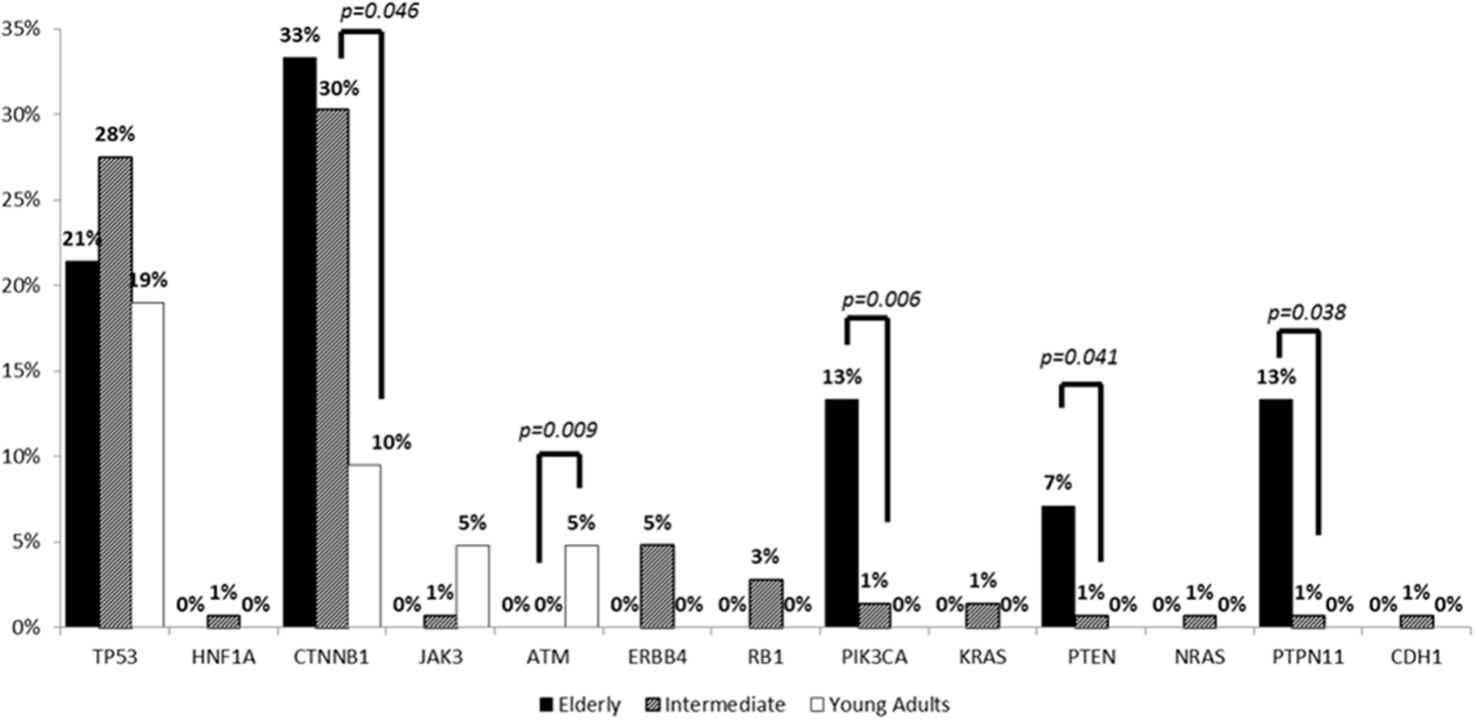
Comparison of pathogenic and presumed pathogenic alterations across the age groups

A total of 47 genes were analyzed for well-characterized hotspots and their respective alteration frequencies and distribution across the different age groups are shown in Figure 4. Differences in the frequency of pathogenic and presumed pathogenic variants are highlighted in Table 2. *TP53* mutations were the most common alteration observed in young adults, but their frequency did not differ significantly amongst the age groups. *CTNNB1* mutations were significantly less frequent in young adults (2/21, 10%) than in intermediate age (44/145, 30%, p=0.046) and elderly (5/15, 33%, p>0.050) patients. Elderly patients exhibited a higher frequency of alterations in PIK3CA/Akt/mTOR pathway components *PIK3CA, PTEN and PTPN11* (7-13%) compared to intermediate age and young adult patients (0-1%). These differences were not significant, though the proportion of elderly and young adult patients tested was much smaller than the intermediate age cohort. Alterations in *JAK3, ATM, ERBB4, KRAS* and *NRAS* were also present and did not differ statistically amongst the groups.

**Table 2:** Frequencies of pathogenic and presumed pathogenic alterations in different age groups

	Young Adult (18-39 years)		Intermediate age (40-74 years)		Elderly (≥ 75 years)		YA vs IA	YA vs E	IA vs E
	N+/N total	%	N+/N total	%	N+/N total	%	p-value	p-value	p-value
**TP53**	4/21	19	39/142	28	3/14	21	NS	NS	NS
**HNF1A**	0/19	0	1/136	0.7	0/13	0	NS	NS	NS
**CTNNB1**	2/21	10	44/145	30	5/15	33	0.046	NS	NS
**JAK3**	1/21	5	1/146	1	0/15	0	NS	NS	NS
**ATM**	1/21	5	0/144	0	0/15	0	0.008	NS	NS
**ERBB4**	0/21	0	7/145	5	0/15	0	NS	NS	NS
**RB1**	0/21	0	4/143	3	0/15	0	NS	NS	NS
**PIK3CA**	0/21	0	2/141	1	2/15	13	NS	NS	0.006
**KRAS**	0/21	0	2/146	1	0/15	0	NS	NS	NS
**PTEN**	0/21	0	1/142	1	1/14	7	NS	NS	0.041
**NRAS**	0/21	0	1/144	1	0/15	0	NS	NS	NS
**PTPN11**	0/21	0	1/146	1	1/15	13	NS	NS	0.038
**CDH1**	0/21	0	1/146	1	0/15	0	NS	NS	NS

YA = young adult; IA = intermediate age; E = elderly; NS = not significant

The overall frequency of alterations appeared to increase with age. Focusing only on pathogenic and presumed pathogenic alterations, the frequency was significantly lower in young adults (0.38 mutations/case) versus intermediate age (0.71, p=0.012) and elderly patients (0.93, p=0.038).

### Biomarker expression in primary and metastatic sites

Differences in biomarker expression were noted between primary and metastatic HCC sites as shown in Table 3. Across all age groups, p-glycoprotein expression was significantly more frequent in primary HCC than in metastases, whereas TOP2A, thymidylate synthase and TUBB3 expression were more frequent in metastases. There was a significantly higher occurrence of *PTEN* mutations in metastases than in primary HCCs, but the absolute frequencies were low (3.6% and 0%, respectively). Among the three subgroups, only the intermediate age group demonstrated significant differences in biomarkers between primary and metastatic HCC. The same expression patterns seen in the entire study population were also observed in this subgroup, except that there was a higher incidence of PIK3CA mutations in metastases (4.5%) than in primary HCC (0%).

**Table 3:** Biomarker expression and tumor genotype in primary HCC and metastatic sites

	Significant Biomarkers	Mets. Vs. Primary	Percent	p values
**Entire study group**	IHC-p-glycoprotein	61/93 vs. 155/199	66% vs. 78%	0.029
	IHC-TOP2A	52/99 vs. 96/248	52% vs. 39%	0.016
	IHC-thymidylate synthase	47/146 vs. 61/254	32% vs. 24%	<0.001
	IHC-TUBB3	11/69 vs. 11/151	16% vs. 7.3%	0.045
	NGS-PTEN	2/55 vs. 0/123	3.6% vs. 0	0.032
**Young adults**		None		
**Intermediate age**	IHC-p-glycoprotein	49/73 vs. 121/152	67% vs. 80%	0.048
	IHC-TOP2A	44/79 vs. 74/195	56% vs. 38%	0.006
	IHC-thymidylate synthase	39/86 vs. 45/199	45% vs. 3%	<0.001
	IHC-TUBB3	10/58 vs. 8/177	17% vs. 4.5%	0.031
	NGS-PIK3CA	2/44 vs. 0/99	4.5% vs. 0	0.032
**Elderly**		None		

## DISCUSSION

In this retrospective series, differences in demographic and molecular features of HCC patients across different age groups were detected. We observed that young adults were more frequently female, residing in Western and Midwestern states, and less frequently expressed the AR, drug resistance and pro-angiogenic proteins compared to older patients. *CTNNB1* mutations, one of the most common pathogenic alterations in HCC, were significantly less common in young adults than in patients 40 years and older. Elderly HCC patients exhibited increased PI3K/Akt/mTOR pathway alterations compared to younger patients.

From a therapeutic perspective, these data suggest the potential for differential sensitivities amongst the subgroups to various agents. Increased MGMT and SPARC expression in patients ≥ 40 years’ old suggests that they might be less responsive to alkylating agents, but more sensitive to taxanes or fluoropyrimidines. SPARC overexpression has been shown to resensitize HepG2 cells to 5-fluorouracil [16]. Intermediate age and elderly patients might also be susceptible to AR inhibitors. The higher incidence of PI3K/Akt/mTOR pathway mutations in elderly patients may predict greater sensitivity to agents targeting this cascade compared to younger patients. However, the increased expression of MRP-1 in patients ≥ 40 years' old may represent an adaptive response to a greater lifetime cumulative exposure to xenobiotics [17] that renders their disease more resistant to treatment than in their younger counterparts.

Differences in therapeutic sensitivities may also exist between primary tumors and metastases. The frequency of p-glycoprotein expression observed in primary HCCs is compatible with the existing literature [18] and is thought to underlie the resistance of these tumors to chemotherapy. Increased expression of TOP2A, thymidylate synthase, TUBB3 and mutations in *PIK3CA* and *PTEN* in HCC metastases suggest that they may be less responsive to 5-fluorouracil and taxanes, but more sensitive to anthracyclines and mTOR inhibitors. Although the predictive clinical value of these biomarkers is limited as cytotoxic chemotherapy is not routinely used to treat HCC, and the mTOR inhibitor everolimus was not active in a phase III trial [19], the different expression patterns nevertheless support the hypothesis that there may be age-specific and tumor site-specific molecular differences in HCC.

We observed a statistically significant increase in the frequency of pathogenic and presumed pathogenic mutations with increasing age. This is different from tumor mutation load (TML) which is thought to correlate with immunogenicity. TML values calculated from somatic missense mutations using the 592 gene panel were unavailable for vast majority of the cases investigated in the current analysis. From a separate study with 98 tumors sequenced with 592-gene panel, we observed TML values to be 5.5 mutations/megabase in the young adults group (n=6), 7.6 in the intermediate group (n=73) and 6.7 in the elderly group (n=18), with no statistical significant differences seen amongst the groups. Given recent evidence of nivolumab activity in HCC [20], the relationship between TML, PD-L1 expression and response to checkpoint inhibitors is of interest. PD-L1 expression was assessed in some tumors and was found to be absent in young adults. Testing in a larger proportion of patients from extremes of the age-spectrum will be necessary to determine if there are age-specific differences in PD-L1 expression.

Our results also suggest etiologic associations and demographic features of young adult and older HCC patients that are compatible with the existing literature. Increased MGMT expression in patients ≥ 40 years' old may reflect lower *MGMT* methylation levels which have been linked with hepatitis C and non-alcohol associated HCC [21]. The higher incidence of *CTNNB1* mutations in this age group also suggests that they are less likely to have hepatitis B induced HCC [22]. However, increasing tumor AR expression with advancing age may indicate a link with the hepatitis B virus given evidence of a positive feedback loop with AR signaling [23]. The higher frequency of PI3K/Akt/mTOR pathway mutations in elderly patients suggests that they are more likely to be of Western than Asian origin [24]. In contrast, *TP53* mutations - the most common alteration observed in young adults - are more frequent in Asian than in Western populations, and are strongly associated with aflatoxin exposure and hepatitis B [22, 24]. Differences in the variables that drive hepatocarcinogenesis in different age groups, and their temporal and spatial interplay with AR signaling, viral status and sex remain to be elucidated.

While prior studies have reported that elderly HCC patients are more likely to be female, possibly as a result of the longer life expectancy of women [9, 12, 13, 15], a decreased female prevalence in patients ≥ 75 years was observed in this cohort. Since many of the studies reporting an increased female to male ratio among elderly HCC patients were conducted in East Asia, the discrepant findings of our study may be partly explained by geographic and racial or ethnic differences. Due to the unavailability of racial and ethnic information, we are unable to determine whether sex ratio differs by race and ethnicity within our Western study cohort.

Weaknesses of this study include the absence of clinical, race or ethnicity data which prevents us from confirming or refuting our assumptions about disease etiology based on the molecular findings, as well as commenting on differences in disease characteristics and oncologic outcomes between the age groups. In addition, the proportion of young adult and elderly patients is small compared to the intermediate age group which encompasses a broad and heterogeneous age range. These differences might have masked other molecular differences between and within the groups. The small number of cases analyzed for some biomarkers also limits the robustness of the comparisons and the conclusions that can be drawn. Given the absence of information on patients’ treatment histories and the timing of specimen collection in relation to specific agents or modalities, we are unable to account for the potential confounding effect of therapy on biomarker expression. Furthermore, the panel of genes tested is not an exhaustive list of all known contributors to hepatocarcinogenesis and maintenance; for example, genetic aberrations affecting chromatin remodeling, telomere maintenance and oxidative stress [25] were not included. Rather, the genes included were selected for their known prognostic and/or therapeutic significance.

In summary, multiplatform profiling data reveals molecular distinctions between young adult and older HCC patients, highlighting potential differences in therapeutic targets amongst the groups. To our knowledge, these data represent one of the only studies to analyze age-specific molecular profiles in HCC, and provide a basis for further exploration and validation of these findings with respect to their clinical and therapeutic implications.

## MATERIALS AND METHODS

Gene sequencing, amplification and protein expression data from anonymized HCC specimens submitted to Caris Life Sciences between 2009 and March of 2016 were retrospectively reviewed. H&E slides were prepared for each tumor sample and were reviewed by board-certified pathologists to verify the diagnosis of HCC on the pathology reports accompanying the tumor samples. Fibrolamellar liver carcinoma, hepatoblastoma and hepatosarcoma were excluded. Not all the same tests were performed on all tumors investigated, due to the different tests requested by treating physicians for each patient, or due to the advancement of testing technologies over time. For NextGen sequencing, tumors tested in 2015 or earlier were sequenced with MiSeq platform while those tested in 2016 or later were sequenced with NextSeq platform. Because patients consented to tumor specimen submission and molecular profiling but not access to their medical records, only basic demographic information was available. Patients were stratified into young adult (18-39 years’ old), intermediate age (40-74 years’ old) and elderly (≥ 75 years’ old) subgroups. For patients living in the United States, region of residence was determined by zip code. The Chi-square test was used for statistical comparisons.

Immunohistochemistry (IHC) was performed on 408 tumors on full formalin-fixed paraffin-embedded (FFPE) sections on glass slides. Slides were stained using an automated system (Benchmark, Ventana Medical Systems, Tucson, AZ; Autostainer, DAKO, Carpinteria, CA) as per the manufacturer's instructions, and were optimized and validated per CLIA/CAO and ISO requirements. Tumor cells were scored for all proteins of interest with the exception of PD-1, which was scored on tumor infiltrating lymphocytes. Staining was scored for intensity (0 = no staining; 1+ = weak staining; 2+ = moderate staining; 3+ = strong staining) and percent of cells staining positive for the protein (0-100%). The primary antibody used against PD-L1 was SP142 (Spring Biosciences). The staining was regarded as positive if its intensity on the membrane of the tumor cells was >=2+ and the percentage of positively stained cells was >5%. Results were categorized as positive or negative by defined thresholds specific to each marker, based on published clinical literature that associates biomarker status with patient responses to therapeutic agents. A board-certified pathologist evaluated all IHC results independently.

Next-generation sequencing (NGS) was performed on 182 tumors on genomic DNA isolated from FFPE tumor samples using either the MiSeq (N=154) platform or the NextSeq (N=28) platform (Illumina, Inc., San Diego, CA). No matched normal tissue was sequenced. For tumors tested with MiSeq, specific regions of the genome were amplified using the Illumina TruSeq Amplicon Cancer Hotspot panel. For tumors tested with NextSeq, a custom-designed SureSelect XT assay was used to enrich 592 whole-gene targets (Agilent Technologies, Santa Clara, CA). All variants were detected with > 99% confidence based on allele frequency and amplicon coverage with an average sequencing depth of coverage of >500 and with an analytic sensitivity of 5%. Genetic variants identified were interpreted by board-certified molecular geneticists and categorized as ‘pathogenic,’ ‘presumed pathogenic,’ ‘variant of unknown significance,’ ‘presumed benign,’ or ‘benign,’ according to the American College of Medical Genetics and Genomics (ACMG) standards. When assessing mutation frequencies of individual genes, ’pathogenic,’ and ‘presumed pathogenic, were counted as mutations while ‘benign’ or ‘presumed benign’ variants and ‘variants of unknown significance’ were excluded.
